# Acute Kidney Injury After Percutaneous Coronary Intervention Guided by Intravascular Ultrasound

**DOI:** 10.7759/cureus.57164

**Published:** 2024-03-29

**Authors:** Minh Tran Duc, Thai Nguyen Quoc, Bach Yen T Nguyen, Ngoc Vu Quang, Nhuong Nguyen Duc, Hung Nguyen Duc, Lam Truong Hoai, Vu Nguyen Hoai, Hung Phan Kieu, Hieu Nguyen Trung

**Affiliations:** 1 Cardiology, Tam Anh Hospital, Ha Noi, VNM; 2 C4 Department, Vietnam National Heart Institute, Ha Noi, VNM; 3 Anesthesiology and Critical Care, Tam Anh Hospital, Ha Noi, VNM

**Keywords:** ivus-guided pci, iodinated contrast, intravascular ultrasound (ivus), primary percutaneous coronary intervention (pci), contrast-induced acute kidney injury

## Abstract

Purpose

We investigated the impact of intravascular ultrasound guidance on reducing the incidence of contrast-induced acute kidney injury (CI-AKI) in patients undergoing percutaneous coronary intervention (PCI).

Methods

Ninety-nine patients were enrolled in this prospective cohort who were not randomly assigned to angiography-guided percutaneous coronary intervention or intravascular ultrasound-guided percutaneous coronary intervention. The patients were hospitalized at the Vietnam National Heart Institute - Bach Mai Hospital between 2019 and 2020. Acute kidney injury incidence during hospitalization was the primary endpoint.

Results

A total of 99 patients were divided into two groups: the intravascular ultrasound-guided group (33 participants) and the angiography-guided group (66 participants). The mean ± SD contrast volume of each group was 95.2 ± 37.1 mL and 133.0 ± 36.0 mL for the ultrasound-guided and angiography-guided groups, with P < 0.0001. Intravascular imaging-guided percutaneous coronary intervention (IVUS-guided PCI) was associated with reduced acute kidney injury incidence during hospitalization: 0.0% vs. 12.12% and P = 0.049.

Conclusions

Intravascular ultrasound is a safe imaging tool that guides percutaneous coronary intervention and significantly reduces the rate of acute kidney injury compared to angiography alone. Patients who have a high chance of experiencing acute kidney injury benefit from using intravascular ultrasound.

## Introduction

After percutaneous coronary intervention (PCI), the rate of contrast-induced acute kidney injury (CI-AKI) was 7.1%, and the incidence of hemodialysis was requested in 0.3% of cases [1]. The Acute Kidney Injury Network recognizes CI-AKI when one or more of the following criteria are present: serum creatinine to 1.5-2 × baseline, ≥0.3 mg/dL absolute serum creatinine increase within 48 hours, or urine output <0.5 mL/kg/h for 6-12 hours [2]. This condition leads to an increase in mortality, treatment time, and hospital costs [3,4]. The development of CI-AKI is influenced by older age, diabetes mellitus, anemia, acute coronary syndrome, cardiogenic shock, congestive heart failure, or left ventricular ejection fraction <45%, baseline renal impairment, and volume of contrast [5,6].

Contrast agents have direct toxicity towards renal tubular epithelial cells, which causes necrosis or loss of function and accelerates the natural death process [7]. Furthermore, contrast agents induce indirect toxicity via chemical mediators like nitric oxide, prostaglandin, endothelin, and adenosine, which leads to vasomotor disorders and ischemia [7].

In 2009, a meta-analysis including 2763 subjects from 16 clinical trials found that iodixanol did not reduce CI-AKI compared with low-osmolar contrast media [8]. In another meta-analysis of data from ten clinical trials with 2839 patients, iso-osmolar contrast media had no significant benefit when compared with low-osmolar contrast media [9].

Reducing the amount of contrast agent used may reduce CI-AKI after coronary intervention. A study of 58,957 patients after coronary intervention showed that contrast volume above the estimated glomerular filtration rate (CV/eGFR) ≥ 2.0 increased CI-AKI with an odds ratio of 1.7 [10]. The European Society of Cardiology has recommended using the minimum amount of contrast agent with a threshold allowing CV/eGFR < 3.7 [11].

Intravascular ultrasound can be used to guide and replace some steps in the intervention process, which is one of the measures to help reduce the amount of contrast [12]. In 2010, Nayak et al. reported the first series of four clinical cases using intravascular ultrasound as a tool to guide intervention, with the advantage of reducing the amount of contrast used to <15 mL [13]. The MOZART randomized clinical trial showed that intravascular ultrasound-guided percutaneous coronary intervention (IVUS-guided PCI) was able to significantly reduce the amount of contrast material to 20.0 mL compared with the conventional intervention group (angiography-guided PCI), which was 64.5 mL (p < 0.001) [14]. The MINICON study showed that the rate of acute kidney injury in the angiography-guided PCI group was 15.0% higher than the IVUS-guided PCI group, which was only 2.0% with p = 0.001 [15].

In this study, we investigated the impact of IVUS guidance in reducing the incidence of CI-AKI during hospital stay in patients undergoing PCI, compared with angiographically guided PCI.

## Materials and methods

This prospective cohort study was conducted at the Vietnam National Heart Institute - Bach Mai Hospital from January 2019 to December 2020. This study complies with the Declaration of Helsinki and was approved by the ethics committee of the hospital. We considered enrolling patients who were 18 years of age or older, underwent PCI, and had one or more of the following criteria associated with the inclusion of patients who were at high risk of CI-AKI: age >75 years, diabetes, anemia, acute coronary syndrome, cardiogenic shock, congestive heart failure, left ventricular ejection fraction <45%, creatinine clearance <60 mL/min/1.73 m^2^. Exclusion criteria included allergy to contrast agents, unstable renal function before PCI, and use of contrast agents <3 days or other nephrotoxic agents <7 days.

The primary outcome was CI-AKI, which was diagnosed when one of the following criteria was present: serum creatinine to 1.5-2 × baseline, ≥0.3 mg/dL absolute serum creatinine increase within 48 hours, or urine output <0.5 mL/kg/h for 6-12 hours.

We calculated the number of patients using the two-proportion estimated sample size formula. In developing countries, patients' economic conditions and health insurance policies are limited, so we calculated the change in the patient ratio between two groups: the IVUS-guided PCI group and the angiography-guided PCI group, which has gone from 1:1 to 1:2. All patients received intravenous fluids before and after PCI. Contrast agents that have been used include iohexol (Omnipaque), iobitridol (Xenetix), and iopromide (Ultravist). The contrast media used are all low-osmolar. The iLab Ultrasound Imaging System (Boston Scientific Corporation, Marlborough, MA) and the Atlantis SR Pro 40 MHz Imaging Catheter were used to perform IVUS imaging. During an automatic pullback at a speed of 0.5 mm/s, vessels were photographed, and if further analysis was needed, manual pullback was available.

The intervention process was conducted in three stages. In the first stage, we performed a coronary angiogram. Four views were used to evaluate the left coronary artery: LAO cranial, LAO cranial, RAO cranial, and RAO cranial. Three views are used to evaluate the right side: LAO, RAO, and LAO cranial. Each scan used between 2 and 5 mL of contrast medium. We carried out coronary intervention in the second stage. The secondary monitor showed the diagnostic scan results for reference. The IVUS-guided PCI group had a guide wire inserted with little or no contrast. We used non-contrast X-rays to capture IVUS probe placement at proximal, distal, and landing zone reference points. The stent length and diameter were determined by evaluating the lesion length and arterial diameter through IVUS. After expanding the stent, IVUS determined the size of the non-compliant balloon. Contrast injections can be used during intervention to check for suspected complications. In the angiography-guided PCI group, we used a reference monitor during the intervention. In the final stage, we performed an angiogram with two different views to confirm the successful intervention results without any complications.

Statistical analysis was performed with SPSS (IBM SPSS Statistics for Windows, Version 23.0, IBM Corp., Armonk, NY). Variables are presented as mean ± standard deviation (quantitative variables) or as count or percentage (categorical variables). Parametric (Student's t-test) and non-parametric (chi-squared test) methods depend on the variable's type (categorical or quantitative) and its distribution (normal or not). Relationships with a significance level (α) less than 0.05 were considered statistically significant.

## Results

Our study included 99 patients, and they were split into two groups: angiography-guided PCI groups with 66 patients and IVUS-guided PCI groups with 33 patients. Table 1 shows the distribution of age, sex, and risk of CI-AKI, including age >75, diabetes, anemia, acute coronary syndrome, cardiogenic shock, congestive heart failure, left ventricular ejection fraction <45%, and eGFR <60 mL/min/1.73 m^2^. Patients in the two groups were selected non-randomly. Despite this, baseline characteristics were evenly distributed between the two groups.

**Table 1:** The baseline characteristics of patients SD: standard deviation; *chi-squared test (comparison of proportions); **Student’s t-test (comparison of means), P ≤ 0.05 is regarded as significant. IVUS: intravascular ultrasound; PCI: percutaneous coronary intervention; eGFR: estimated glomerular filtration rate.

	Total	Angiography-guided PCI	IVUS-guided PCI	P-value
Male [n, (%)]	66 (66.7)	46 (69.7%)	20 (60.6%)	0.37*
Age [years, mean ± SD]	69.5 ± 8.9	69.7 ± 8.8	69.2 ± 9.3	0.76**
Age >75 [n, (%)]	22 (22.2)	14 (21.2)	8 (22.2)	0.73*
Diabetes [n, (%)]	35 (35.4)	26 (39.4)	11 (33.3)	0.77*
Anemia [n, (%)]	37 (37.4)	24 (36.4)	13 (39.4)	0.72*
Acute coronary syndrome [n, (%)]	94 (94.9)	62 (93.9)	32 (97.0)	0.88*
Cardiogenic shock [n, (%)]	14 (14.1)	10 (15.2)	4 (12.1)	0.78*
Congestive heart failure or left ventricular ejection fraction <45% [n, (%)]	39 (39.4)	23 (34.8)	16 (48.5)	0.84*
Serum creatinine [µmol/L, mean ± SD]	100.3 ± 41.1	98.8 ± 36.0	103.1 ± 50.2	0.86**
eGFR [mL/min/1.73 m^2^, mean ± SD]	66.7 ± 23.3	67.1 ± 21.9	65.9 ± 26.3	0.76**
eGFR < 60 mL/min/1.73 m^2 ^[n, (%)]	47 (47.5)	29 (43.9)	18 (54.5)	0.32*

The angiographic characteristics of the patients included in the study did not show any statistical differences (Table 2). The number of stents, stent diameter, total sum of stent length, and fluoroscopic time in the IVUS-guided PCI group tended to be higher than those in the angiography-guided PCI group, but the difference was not statistically significant. We did not record any cases of dissection or slow flow/no flow after intervention in the two groups.

**Table 2:** Angiographic characteristics SD: standard deviation; *chi-squared test (comparison of proportions); **Student’s t-test (comparison of means); P ≤ 0.05 is regarded as significant. IVUS: intravascular ultrasound; PCI: percutaneous coronary intervention; eGFR: estimated glomerular filtration rate; LM: left main; LAD: left anterior descending artery; LCx: left circumflex artery; RCA: right coronary artery; ACC: American College of Cardiology; AHA: American Heart Association.

	Total	Angiography-guided PCI	IVUS-guided PCI	P-value
Femoral access [n, (%)]	17 (17.2)	9 (13.6)	8 (24.2)	0.19*
Treated vessel				
LM [n, (%)]	3 (3.0)	3 (4.6)	0 (0.0)	0.55*
LAD [n, (%)]	53 (53.5)	34 (51.5)	19 (57.6)	0.57*
LCx [n, (%)]	27 (27.3)	17 (25.8)	10 (30.3)	0.63*
RCA [n, (%)]	30 (30.3)	22 (33.3)	8 (24.2)	0.35*
ACC/AHA lesion type B2 or C [n, (%)]	83 (83.8)	55 (83.3)	28 (84.8)	0.85*
Number of stents [number, mean ± SD)]	1.47 ± 0.6	1.39 ± 0.5	1.63 ± 0.7	0.11**
Stent diameter [mm, mean ± SD]	3.07 ± 0.6	3.04 ± 0.8	3.10 ± 0.3	0.35**
Total sum of length stent [mm, mean ± SD]	28.1 ± 3.3	26.9 ± 5.2	28.7 ± 4.4	0.18**
Fluoroscopic time [sec, mean ± SD]	1017 ± 394	997 ± 339	1099 ± 482	0.24**

**Figure 1:**
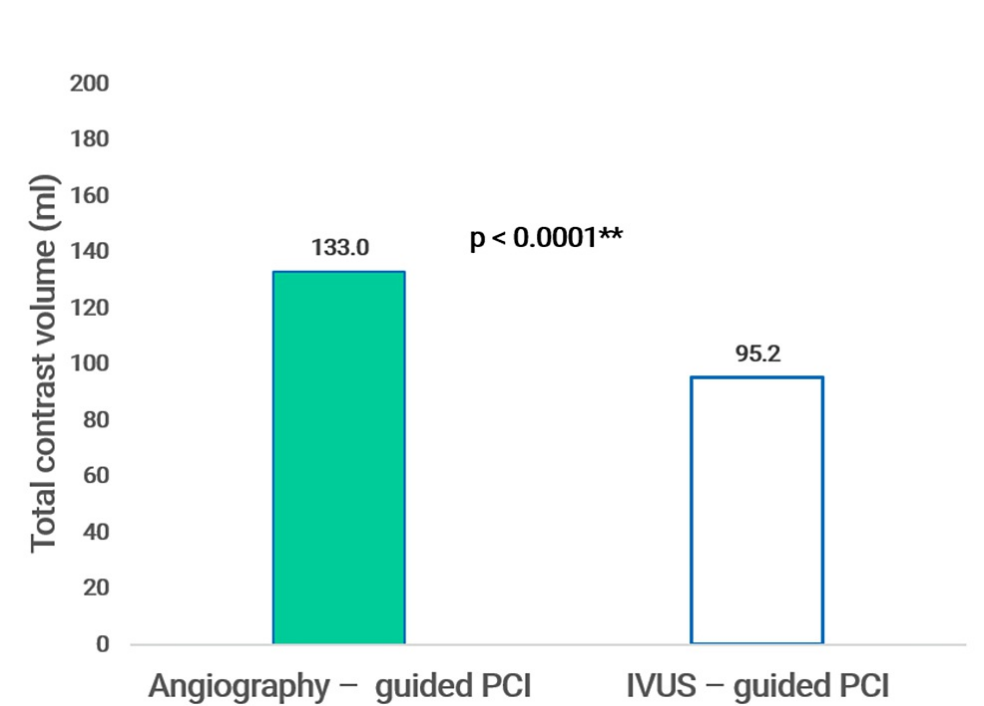
Total contrast volume SD: standard deviation; **Student’s t-test (comparison of means). P ≤ 0.05 is regarded as significant.

The total volume of contrast was 133.0 ± 36.0 mL in the angiography-guided group vs. 95.2 ± 37.1 mL in the IVUS-guided group, with a P-value < 0.0001 (Figure 1).

**Figure 2:**
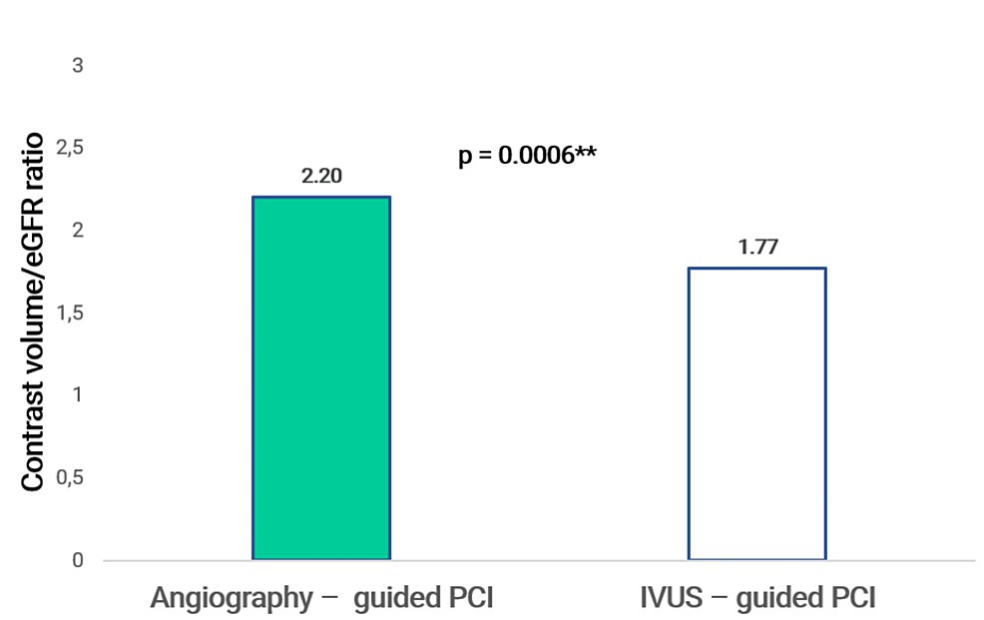
Contrast volume/eGFR ratio SD: standard deviation; **Student’s t-test (comparison of means). P ≤ 0.05 is regarded as significant.

The two groups had a significant difference in the CV/eGFR ratio, 2.20 ± 0.98 vs. 1.77 ± 1.32, and P = 0.0006 (Figure 2). Additionally, we discovered that the CV/eGFR ratio ≥2.0 in group IVUS-guided PCI was considerably less than that in group angiography-guided PCI (21.2% vs. 54.5%, p = 0.001).

Table 3 shows the clinical outcomes during hospitalization. Angiography-guided PCI was diagnosed with CI-AKI in 12.1% of patients treated, while IVUS-guided PCI was diagnosed with CI-AKI in 0.0%, P = 0.049. Only one patient died in this study despite receiving dialysis. Stent thrombosis, myocardial infarction, or revascularization intervention were not present in any cases.

**Table 3:** Clinical outcomes *Chi-squared test (comparison of proportions). P ≤ 0.05 is regarded as significant.

	Angiography-guided PCI	IVUS-guided PCI	P-value
Acute kidney Injury [n, (%)]	8 (12.1)	0 (0.0)	0.049*
Renal replacement therapy [n, (%)]	1 (1.5)	0 (0.0)	-
Death [n, (%)]	1 (1.5)	0 (0.0)	-
Stent thrombosis, myocardial infarction, or unplanned revascularization [n, (%)]	0 (0.0)	0 (0.0)	-

## Discussion

The results of our study show that the average amount of contrast agent used in the IVUS-guided PCI group was 95.2 ± 37.1 mL, significantly lower than that of the angiography-guided PCI group, 133.0 ± 36.0 mL, with p < 0.0001. Similar results were reported in the MOZART trial: IVUS-guided intervention reduced the amount of contrast agent to an average of only 20.0 mL compared to 64.5 mL in the conventional intervention group with p < 0.001 [14]. In the MINICON study by Sakai et al., the contrast reduction was even more spectacular, with an average of 22.0 mL in the intervention group under IVUS guidance compared to the conventional intervention group of 130.0 mL with p < 0.0001 [15].

The MOZART study allowed the intervention to be divided into two phases in some patients, with the first only diagnostic imaging and the second reperfusion intervention >72 hours apart [14]. The MINICON study had 100% of patients treated with such a procedure, and the amount of contrast used was calculated in the second phase [15]. We did not design a similar process because local health insurance policies did not allow it. Our study calculated the total contrast volume of the entire process, including three stages. Therefore, the amount of contrast agent used in these two studies was less than in our study.

In the past few years, one study using the DyeVert system was able to reduce the amount of contrast used from 112 ± 85 mL to 67 ± 51 mL, p < 0.0001 [16]. This is a device capable of collecting the excess amount of contrast that will flow from the coronary artery into the aorta at each injection of contrast based on the pressure change through the one-way valve to the reservoir [16]. A similar system called Avert has also had positive results [17]. In the near future, the combination of these systems with IVUS-guided interventions will aid in further reducing the amount of contrast required.

In the angiography-guided PCI group, eight cases were diagnosed with CI-AKI (12.1%), while in the IVUS-guided PCI group, there were no cases. With a P-value below 0.05, this difference was statistically significant. Previous MOZART study data revealed that the IVUS-guided PCI group CI-AKI results were 7.3% less than the angiography-guided PCI group, which was 19.0% [14]. However, the statistical significance of this result was not reached when P > 0.05. The MINICON study revealed that the rate of CI-AKI in the IVUS-guided PCI group versus the conventional intervention group was significantly decreased from 15.11% to 2.04% with P = 0.001 [15]. Our study achieved similar beneficial results as the MINICON study. We found that IVUS-guided significantly reduced the CV/eGFR ratio, 1.77 ± 1.32, compared to 2.20 ± 0.98 in the conventional intervention group, with a P-value of 0.0006. This helped the CV/eGFR ratio ≥2.0 in the IVUS-guided PCI group to be significantly lower than the angiography-guided PCI group, 21.2% vs. 54.5%, with P = 0.001. It is suggested by this result that achieving a CV/eGFR ratio below 2.0 should be the goal to lower the rate of CI-AKI after coronary intervention.

Our study has a few limitations. It was not randomized, so selection bias cannot be eliminated. Our country faces a significant obstacle in conducting randomized studies in these high-risk patient groups. Finally, this study focused on CI-AKI only during the hospital stay; cases that occurred after discharge were not analyzed.

## Conclusions

Using intravascular ultrasound as an imaging tool is safe and helps guide percutaneous coronary intervention, resulting in a significant reduction in contrast volume and the rate of acute kidney injury compared to angiography alone. In patients who have a high chance of experiencing contrast-induced acute kidney injury (including age >75 years, diabetes, anemia, acute coronary syndrome, cardiogenic shock, congestive heart failure, or left ventricular ejection fraction <45%, creatinine clearance <60 mL/min/1.73 m^2^), intravascular ultrasound-guided percutaneous coronary intervention may be beneficial.
